# Coil Embolization and Endoscopic Hematoma Removal for Ruptured Cerebral Aneurysm With Intracranial Hematoma Under Local Sedation: A Case Report

**DOI:** 10.7759/cureus.73663

**Published:** 2024-11-14

**Authors:** Taigen Sase, Homare Nakamura, Hirobumi Nakayama, Gaku Hidaka, Kiyotaka Wakatsuki

**Affiliations:** 1 Department of Neurosurgery, St. Marianna University School of Medicine, Yokohama Seibu Hospital, Yokohama, JPN

**Keywords:** coil embolization, dexmedetomidine hydrochloride, endoscopic surgery, local anesthesia, ruptured aneurysm

## Abstract

Intracerebral hematomas (ICHs) can complicate ruptured cerebral aneurysms. The standard approach for these cases has traditionally involved craniotomy with clipping and hematoma evacuation. Recently, however, a combination of coil embolization and neuroendoscopic hematoma removal has shown promise. We report a case of a ruptured internal carotid artery aneurysm with ICH successfully treated using coil embolization and neuroendoscopic hematoma removal under intravenous sedation. A woman in her late 70s presented to our hospital with severe consciousness disturbance and was diagnosed with subarachnoid hemorrhage (SAH) and an intratemporal hematoma caused by a ruptured aneurysm at the left internal carotid-posterior communicating artery bifurcation. Her condition was complicated by low cardiac output and hypotension due to Takotsubo cardiomyopathy, making general anesthesia unfeasible. Coil embolization was performed the same day under local sedation with dexmedetomidine hydrochloride. The next day, we used a neuroendoscope to evacuate the intratemporal hematoma under local anesthesia. Despite the severity of the SAH, the patient survived and was later transferred to a long-term care hospital. This approach appears effective for patients with ruptured cerebral aneurysms and ICH who are not suitable candidates for general anesthesia.

## Introduction

Subarachnoid hemorrhage (SAH) with intracerebral hematoma (ICH) occurs in approximately one-third of all SAH cases [1-3], often resulting in poor prognosis and high mortality rates [4,5]. Traditionally, craniotomy with clipping has been the standard approach for SAH with ICH, allowing both aneurysm repair and hematoma evacuation. Recently, favorable outcomes have been reported with coil embolization followed by craniotomy for hematoma removal. Additionally, combining coil embolization with endoscopic intraventricular hematoma (IVH) removal has shown promise in patients with both SAH and IVH, typically managed under general anesthesia.

In this case, we treated a patient presenting with SAH due to a ruptured aneurysm at the left internal carotid-posterior communicating artery bifurcation, accompanied by a left intratemporal hematoma. Given the patient’s compromised general condition, making general anesthesia unfeasible, we performed coil embolization of the aneurysm and endoscopic hematoma removal under local anesthesia with intravenous sedation. Herein, we describe the surgical technique and perioperative management for this approach.

## Case presentation

**Figure 1:**
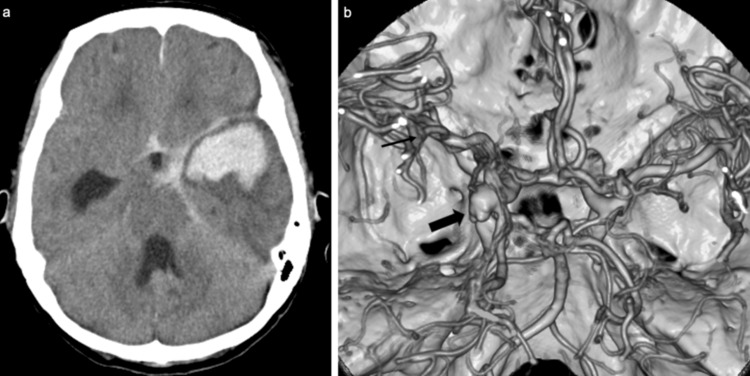
Preoperative CT and CTA images (a) Axial CT scan showing diffuse SAH and left intratemporal cerebral hemorrhage. (b) CTA indicating the ruptured aneurysm at the left internal carotid-posterior communicating artery bifurcation (maximum diameter, 6.7 mm; arrow). CTA, CT angiography; SAH, subarachnoid hemorrhage

A 79-year-old woman weighing 34 kg, with a history of pulmonary tuberculosis and an unruptured cerebral aneurysm, was transported to another hospital with a disturbance in consciousness. Plain head CT revealed a diffuse SAH and a left intratemporal hematoma (Figure 1a), and the patient was subsequently transferred to our hospital for further management. Upon arrival, the patient was comatose (Glasgow Coma Scale (GCS) E1V1M4) with anisocoria, showing a pupil diameter of 2 mm on the right and 3 mm on the left, with no light reflex on the left. Contrast-enhanced CT angiography demonstrated a 6.7 mm aneurysm at the left internal carotid posterior communicating artery bifurcation (Figure 1b), and the volume of the intratemporal hematoma was 35.2 ml. The patient was diagnosed with SAH, World Federation of Neurological Surgeons (WFNS) clinical grade V, due to a ruptured aneurysm at the left internal carotid posterior communicating artery bifurcation.

At the time of admission, the patient’s systolic blood pressure was <90 mmHg. Cardiac ultrasound revealed reduced cardiac function with an ejection fraction of approximately 20%, and electrocardiography showed ST changes. Blood tests indicated a brain natriuretic peptide (BNP) level of 1606.2 pg/ml, suggesting cardiac stress and Takotsubo cardiomyopathy. After consultation with an anesthesiologist, general anesthesia was deemed challenging. Although the patient’s GCS was E1V1M4, her respiratory status did not necessitate endotracheal intubation. Given the patient’s severe SAH and the family’s request for aggressive treatment, we decided to proceed with acute intervention.

**Figure 2:**
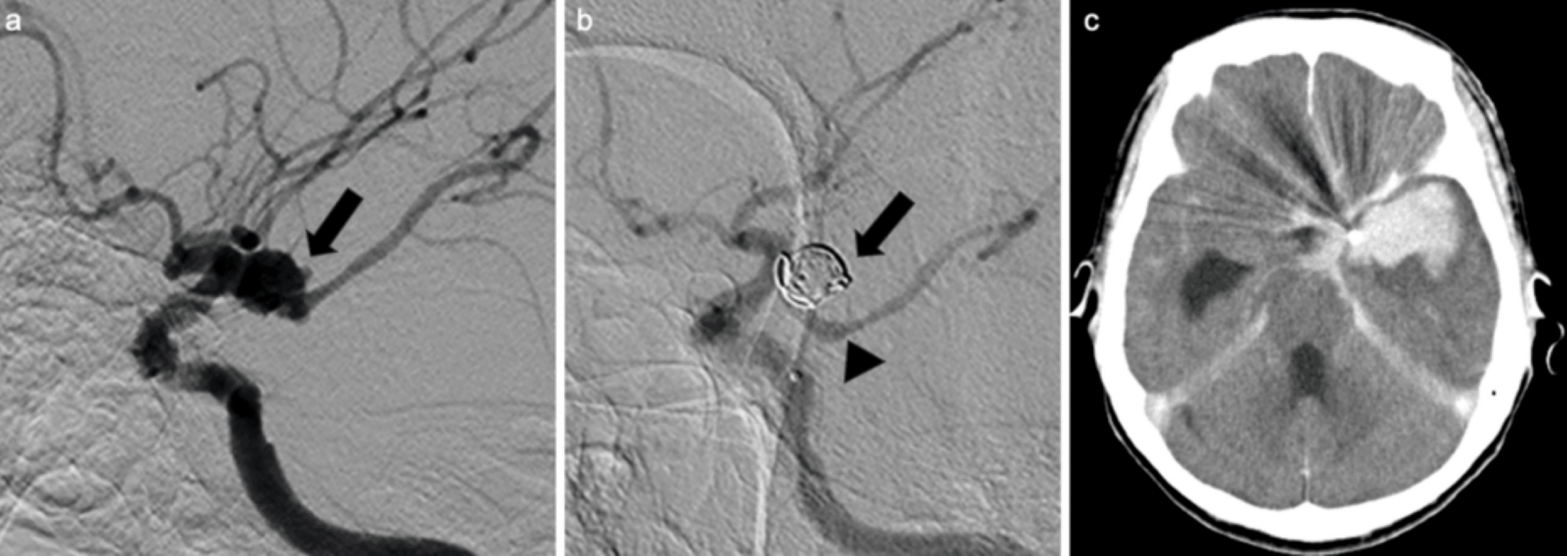
Coil embolization procedure (a) Angiography (left anterior oblique view) before coil embolization showing an aneurysm at the left internal carotid-posterior communicating artery bifurcation with a bleb (arrow). (b) Angiography (left anterior oblique view) after coil embolization confirming successful embolization within the aneurysm (arrow), with maintained blood flow through the posterior communicating artery (arrowhead). (c) CT scan immediately following coil embolization showing no increase in the intratemporal hematoma.

We planned to perform coil embolization under local anesthesia and intravenous sedation using dexmedetomidine hydrochloride. The right femoral artery was punctured, and an 8.2-Fr long sheath was placed. An 8-Fr FlowGate catheter (Concentric Medical, Inc., Stryker Neurovascular, Fremont, USA) was guided and placed in the cervical portion of the left internal carotid artery (Figure 2a). After placing the guiding catheter, 3,000 units of heparin sodium were administered intravenously. A 4.2-Fr FUBUKI catheter (Asahi Intecc Co., Ltd., Seto, Japan) was placed in the petrous portion of the left internal carotid artery to provide support during coil embolization. Using a CHIKAI 0.014 inch 200 cm catheter (Asahi Intecc Co., Ltd.), an Excelsior SL-10 (Concentric Medical, Inc.) was guided into the aneurysm. An Axium Prime Frame (5 mm × 10 cm; Micro Therapeutics Inc., Irvine, California, USA) was placed through the microcatheter into the aneurysm, followed by sequential filling with AXIUM PRIME coils (Micro Therapeutics Inc.). The aneurysm was completely embolized with an AXIUM PRIME Helix 1 mm × 3 cm coil (Micro Therapeutics, Inc.) as a finishing coil. The aneurysmal neck plastic balloon was not used during the procedure. The final angiogram showed preservation of blood flow in the left posterior communicating artery, and the aneurysm was well embolized (Figure 2b). The surgical time was 65 minutes. Post-embolization CT scans showed no significant increase in the volume of the intratemporal hematoma (Figure 2c). Anticoagulant therapy used during the endovascular procedure was not reversed, and no changes in anisocoria were observed immediately post-surgery.

**Figure 3:**
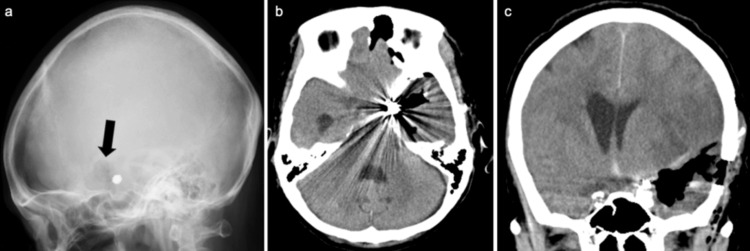
X-ray and CT following endoscopic hematoma evacuation (a) Lateral X-ray showing the burr hole location (arrow). (b, c) Axial and coronal CT scans confirming successful hematoma removal.

On the day after the endovascular surgery, blood tests showed that coagulation function had normalized, and BNP levels decreased to 1,013.0 pg/ml. Cardiac ultrasound indicated improved cardiac function with an ejection fraction of approximately 40%. We proceeded with endoscopic hematoma evacuation under local anesthesia and intravenous sedation using dexmedetomidine hydrochloride. The patient was positioned supine with the left shoulder elevated using a pillow and the head rotated to the right, secured in a horseshoe-shaped head fixation device. A burr hole was made 3 cm anterior and 3 cm superior to the external auditory canal to reach above the hematoma. After local subcutaneous infiltration anesthesia, a 4 cm skin incision was made, and the temporal muscle was incised to create the burr hole (Figure 3a). The dura was incised, the hematoma cavity was punctured, and a neurosheath (22-Fr, inner lumen 7.0 mm, outer diameter 8.1 mm, effective length 10 cm, Medikit Co., Ltd., Tokyo, Japan) was inserted into the cavity. A suction tube was used to remove the hematoma, which was visualized using a 2.7 mm, 0-degree rigid neuroendoscope (Olympus Medical Systems Corp., Hachioji, Japan). The hematoma was carefully removed circumferentially. The surgical time was 47 minutes. Postoperative CT scans confirmed no bleeding complications and >90% of the hematoma was removed (Figure 3b, 3c). The postoperative pupil diameter improved to 2 mm on the right and 2.5 mm on the left. A CT scan performed the day after the endoscopic surgery showed no rebleeding and antiplatelet therapy was initiated as required after coil embolization. Electrocardiography showed resolution of ST changes, indicating improvement in Takotsubo cardiomyopathy.

**Figure 4:**
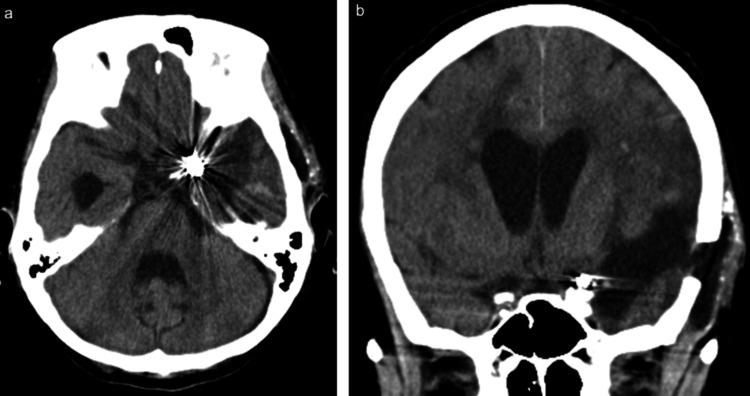
CT taken 20 days after endoscopic hematoma evacuation (a, b) Axial and coronal CT scans showing the post-surgical condition 20 days after hematoma evacuation.

No significant cerebral infarction was observed during the management of cerebral vasospasm. Shunt surgery for hydrocephalus was not required, although mild lateral ventricular enlargement was noted (Figure 4a, 4b). The modified Rankin scale score at 24 days post-onset was 5 (GCS 9), and the patient was transferred to a long-term care facility.

## Discussion

We report a case of a ruptured left internal carotid artery aneurysm with an intratemporal hematoma, treated with a combination of coil embolization and endoscopic hematoma removal under local anesthesia and intravenous sedation using dexmedetomidine hydrochloride. Given the patient’s poor general condition, general anesthesia was deemed challenging, making local anesthesia a reasonable alternative.

SAH with ICH is a severe condition with a poor prognosis [4,5]. Craniotomy clipping has traditionally been the standard procedure for ICH removal. Recently, advances in neuroendovascular techniques have led to increased use of coil embolization for SAH. Coil embolization followed by craniotomy hematoma evacuation has shown good outcomes [4,5]. Additionally, the combination of coil embolization and endoscopic surgery for intracranial hematoma, particularly IVH, has been reported [6,7], generally performed under general anesthesia.

In the present case, coil embolization of the ruptured aneurysm and endoscopic hematoma removal were performed under local anesthesia with intravenous sedation, given the patient’s poor condition. While this approach deviates from standard guidelines, it may be suitable in certain cases, such as the one described here. However, it is important to note that this method requires careful patient selection and is not universally recommended. In cases where respiratory status deteriorates, immediate endotracheal intubation may be necessary.

General anesthesia is the standard treatment for ruptured aneurysms. In the management of ruptured cerebral aneurysms, it is essential to maintain cerebral perfusion pressure while controlling blood pressure to prevent aneurysm re-rupture, and general anesthesia plays a crucial role in achieving this. In Japan, general anesthesia is used for all craniotomy surgeries for ruptured aneurysms and in 85% of endovascular surgeries for ruptured aneurysms [8]. While general anesthesia is necessary to improve angiographic image quality by ensuring patient immobilization, recent reports have highlighted the effectiveness of local anesthesia as well. Coil embolization for ruptured cerebral aneurysms performed under conscious local sedation is safe for low-grade SAH, with a complication rate comparable to that of general anesthesia [9]. In contrast, although the incidence of thromboembolic events after coil embolization for ruptured cerebral aneurysms under local anesthesia is similar to that under general anesthesia, the incidence of intraoperative rupture is significantly higher [10]. However, with appropriate patient selection, endovascular surgery allows for treatment under local anesthesia, an option that is less feasible with craniotomy.

For ICH surgery, general anesthesia is often the preferred method. In Japan, general anesthesia is used in 89% of ICH surgeries [8]. Additionally, over the past three years, endoscopic surgery has been performed in 18% of surgically treated ICH cases [8]. Endoscopic hematoma removal can often be performed under local anesthesia, unlike craniotomy, which requires general anesthesia. However, general anesthesia is usually preferred for endoscopic surgery to ensure safety through immobilization, although endoscopic surgery can be performed under local anesthesia when carefully selected.

Peri- and post-endovascular surgical antithrombotic therapy, including heparinization during surgery and the use of antiplatelet drugs afterward, presents a challenge in combined coil embolization and endoscopic hematoma removal for ruptured cerebral aneurysms with ICH. In this case, systemic heparinization was administered after placing a guiding catheter for endovascular surgery, and the ruptured aneurysm was successfully embolized without any thromboembolic complications. Anticoagulant therapy was not reversed post-procedure due to the absence of rebleeding signs. Coagulation function was normalized the day after endovascular surgery, allowing for the subsequent endoscopic procedure. Antiplatelet drugs were initiated after confirming no postoperative bleeding on CT. The approach to perioperative antithrombotic therapy following coil embolization for ruptured cerebral aneurysms varies among institutions and surgeons. Several considerations remain, such as whether antiplatelet drugs should be administered at the time of coil embolization, whether systemic heparinization should be employed, if it should be neutralized after embolization, and the timing for initiating antiplatelet therapy. The source of bleeding can usually be identified when successful aneurysm embolization is achieved, but bleeding may still occur post-procedure due to the transcortical approach in endoscopic hematoma removal. Therefore, antithrombotic therapy should be managed with caution.

Alternative treatments would not typically be performed during the acute phase on the same day. However, surgery under general anesthesia should be considered once cardiac dysfunction caused by severe SAH improves. If preventing re-rupture during the acute phase is the primary concern, immediate coil embolization of the ruptured aneurysm should take precedence, although careful case selection is necessary.

This patient, with severe SAH, WFNS grade V, and reduced cardiac function, presented a challenge for general anesthesia. The ruptured aneurysm with ICH was successfully treated with combined surgery, using local anesthesia under intravenous sedation in a short surgical time. For cases of SAH with ICH where general anesthesia poses challenges, a surgical procedure under local anesthesia with sedation, as in this case, can be considered. However, this approach deviates from established guidelines and should be carefully explained to the patient’s family.

## Conclusions

We managed a case of SAH due to a ruptured aneurysm at the left internal carotid posterior communicating artery bifurcation, accompanied by a left temporal lobe ICH, using coil embolization and endoscopic hematoma removal under local anesthesia with intravenous sedation. Although careful case selection is essential, this surgical approach can be performed in a short duration and may be appropriate for severe cases.
